# Using geometric criteria to study helix-like structures produced in molecular dynamics simulations of single amylose chains in water†

**DOI:** 10.1039/d1ra00071c

**Published:** 2021-03-24

**Authors:** Mohammad Hassan Khatami, William Barber, Hendrick W. de Haan

**Affiliations:** Ontario Tech University, Department of Physics 2000 Simcoe St N Oshawa ON L1H 7K4. Canada Hendrick.deHaan@ontariotechu.ca

## Abstract

Amylose is a linear polymer chain of α-d-glucose units connected through α(1 → 4) glycosidic bonds. Experimental studies show that in non-polar solvents, single amylose chains form helical structures containing precise H-bond patterns. However, both experimental and computational studies indicate that these perfectly H-bonded helices are not stable in pure water. Nevertheless, amylose chains are observed to form helix-like structures in molecular dynamics (MD) simulations that exhibit imperfect H-bond patterns. In this paper, we study the structure of amylose chains in water using MD simulations to identify and characterize these “imperfect” helical structures. To this end we devise geometry-based criteria to define imperfect helical structures in amylose chains. Using this approach, the propensity of amylose chains to form these structures is quantified as a function of chain length and solvent temperature. This analysis also uncovers both short and long time helix-breaking mechanisms such as band-flips and kinks in the chain. This geometric approach to defining imperfect helices thus allows us to give new insight into the secondary structure of single amylose chains in spite of imperfect H-bond patterns.

## Abstract 2

We introduce a geometrical approach to capture and study helix-like structures in MD simulations of single amylose chains in water.

## Graphical abstract

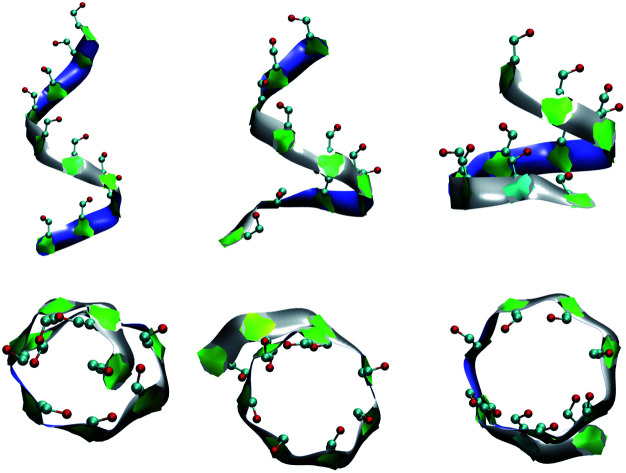

## 1 Introduction

Carbohydrates, along with proteins, lipids, and nucleic acids (*e.g.*, DNA and RNA), are one of the essential macromolecules of biochemistry.^1^ In particular, certain carbohydrates – such as glucose, starch, and glycogen – are the main sources of energy in food. Glucose-containing macromolecules such as glycoproteins and glycolipids also play essential roles in biology.^2^ Further, emerging bio-nanotechnology makes use of glucose-based carbohydrate nanoparticles, such as phytoglycogen, for cosmetic^3^ applications and starch and amylose chains for drug delivery purposes.^4,5^

**Fig. 1:**
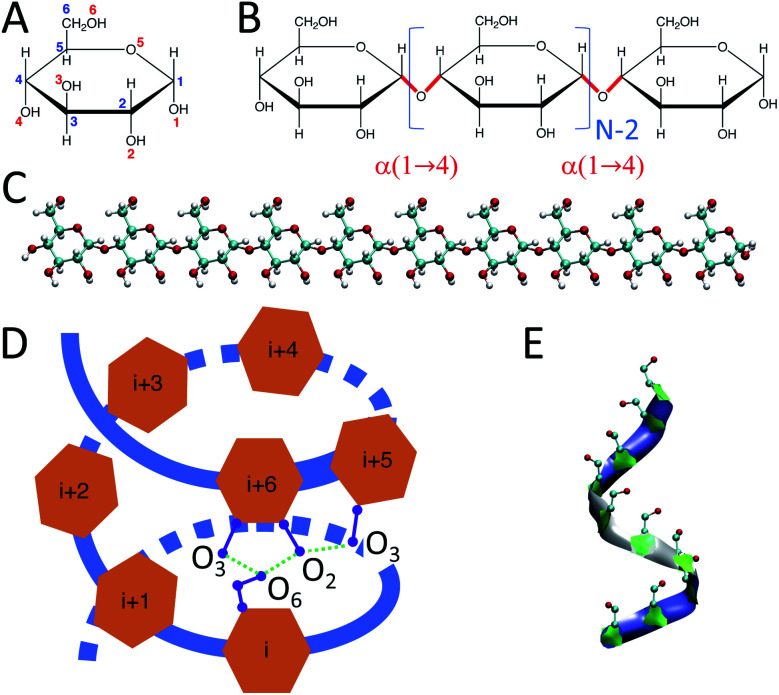
Structure of α-d-glucose as a single monomer and in polymer configurations: (A) structure of a single α-d-glucose unit where blue numbers indicate carbons and red numbers correspond to oxygens; (B) amylose chain of α-d-glucose units connected through α(1 → 4) glycosidic bonds where *N* represents the size of the amylose chain; (C) initially-extended structure of an amylose chain containing 10 glucose units; (D) schematic structure of a perfect H-bonded helix of size 6. For clarity, only selective H-bonds between residues *i* and *i* + 6 and residues *i* + 5 and *i* + 6 are presented; (E) a helix-like structure produced in our MD simulations of an amylose chain of size 10.

Starch is a mixture of two main carbohydrate molecules: amylose chains and amylopectin.^6^ Amylose is a linear polymer of α-d-glucose units connected through α(1 → 4) glycosidic bonds (Fig. 1).^7^

The length of these chains varies between ∼600 to up to ∼18 000 glucose units in nature.^8^ Amylopectin is a branched polymer with α-d-glucose molecules connected through α(1 → 4) and α(1 → 6) glycosidic bonds.^9^ Here, branching occurs every 6 to ∼100 monomers.^10^

Experimental studies have shown that amylose chains make helical structures in single and double-stranded polymorphs.^11^ Double-stranded amylose chains have two main crystal forms, A and B.^12–14^ In both crystal forms, the amylose chain generates left-handed helical structures that produce inter-molecular H-bonds between the two strands of the chain. However, unlike double-stranded amylose, the single-stranded chain (known as V-amylose) forms helical structures only in the presence of iodine, DMSO, alcohols or fatty acids, but not in pure water.^15–20^ As an example, Helbert *et al.*^16^ explored the role of *n*-butanol and *n*-pentanol molecules in the formation of V-type amylose helix. The X-ray crystallography results of single amylose chain structures by Gessler *et al.*^15^ indicate the presence of H-bonds between consecutive glucose residues, where O3 (oxygen atom number 3) of a glucose is bonded to the O2 of the consecutive one (Fig. 1). In addition, there are other intra-molecular H-bonds between the glucose molecules of indices *i* and *i* + 6, *i* and *i* + 7, and *i* and *i* + 8, which represent helices of size 6, 7 and 8 respectively. For example, in the case of a helix of size 6, O6 of residue *i* is connected to O2 and O3 of residue *i* + 6 through H-bonds (Fig. 1-D).

Despite the abundance and the importance of carbohydrates in nature, there are limited number of studies of the structure of amylose chains – particularly in comparison to the vast amount of literature concerning polypeptide structure. Most of the experimental studies that do exist use X-ray crystallography to investigate helical structures. Other possible secondary structures (such as band-flips^15,21^ or protein-like turns), as well as tertiary configurations (a combination of different secondary structures on the same chain) in the amylose chains are less explored.

Experimental studies of single amylose chains in aqueous solution have a long history. Early work employed various experimental methods to probe the helicity of amylose in various solvents. The consensus of this work is that sections of single amylose chains in water rapidly transition between helix and coil structures.^22–27^ Thus, the chain at any point in time is expected to have random helical sections, but these are not long-lasting.

More recently, computer simulations have been employed to probe these same questions. While initial studies gave great insight into the dynamics, they were limited in terms of both the length of the amylose chains and the total time of the simulations.^28–33^ The results were thus difficult to directly compare to previous experimental work examining large chains over long periods. However, a consistent result was that long lasting helical structures – defined by strict H-bonding patterns – were not observed in the simulations.

In the past decade, increases in computational prowess have enabled simulations of longer chains for longer times. Again, studies that relied on strict H-bonding patterns to define helices found that helices were highly unstable in aqueous solutions.^21,34,35^ The lack of stable amylose–amylose H-bonds in water is not surprising as these amylose groups can also form H-bonds with water itself. This particular result is also confirmed *via* NMR and AFM studies.^22,36^ Thus, increasing the hydrophobic nature of the solvent is found to greatly increase the stability of the helices. On the other hand, introducing a smaller concentration of partially hydrophobic molecules can also increase helix stability in water by providing a “core” that the helix can wrap around such that the hydrophobic regions of the amylose chain associate with the hydrophobic molecules and the hydrophilic regions are still satisfied *via* intra-amylose chain H-bonding.^34,35^

Given the complex, amphiphilic nature of amylose chains in water that is suggested from these results, questions of the structure and dynamics of the chains in water remain – particularly with respect to the role of stable H-bonding. Two simulation studies of note have addressed these points by characterizing the chain structures not simply *via* H-bonding patterns, but also by considering the radius of gyration of the chains.^21,37^ Through sufficient generation of data over long enough trajectories, these studies were able to discriminate different amylose structures. In both cases, these are broadly classified as helical or coiled with helical structures exhibiting larger *R*_g_ values. This method of classification also allowed analysis of the transition between these states. While this work was able to give more nuanced insight into the conformation of amylose chains in water, it largely did so by sacrificing detailed quantification of local structures for a more general single metric approach: *R*_g_ of the whole chain.

In this paper, we endeavour to fill the gap between these extremes. A geometric definition of helicity is introduced that does not rely on the overly restrictive H-bond definition but does retain information about local structures. In the remainder of this paper, the term “imperfect-helices” is used to identify these helical structures that are captured with our method. This is in opposition to “perfect” helices defined by precise H-bond patterns. Identifying helix-like conformations gives insight into the structure and dynamics of single amylose chains, which then serves as a basis to study more complex carbohydrate systems such as starch and glycogen/phytoglycogen nanoparticles.

Performing GPU-based simulations, we are able to generate trajectories on the order of microseconds for chains consisting of 10, 20, and 30 glucose units. These long trajectories give ample data to characterize the structure and dynamics of the chains across these sizes. The effect of temperature is also examined. Our proposed criteria for helix identification is shown to be successful in recovering the established picture for amylose chains in water: the chain consists of random sections of helicity with rapid helix to coil transitions. We are also able to correlate this behaviour to chemical details such as the frequency of band-flips as well as larger-scale measurements such as how spontaneously occurring “kinks” in the chain can impact the total potential energy of the molecule and its radius of gyration.

## 2 Methods

### 2.1 System setup and MD simulations

Initially-extended configurations of amylose chains containing *N* = 10, 20, 30 glucose units are used to set up the simulations (Fig. 1-C). These extended structures are built using custom C++ code. The code moves the glucose monomers along the hypothetical line connecting O1 and O4 in a glucose monomer. It places the O1 of the new glucose residue on the O4 of the previous glucose monomer. Then, the code removes the unwanted atoms, *i.e.*, H1 of the new monomer and H4 of the previous one. For the amylose chain of size 10, simulations of 1 μs are conducted across a range of temperatures. Temperatures were chosen to span from close to freezing (280 K) up to close to boiling (360 K) in steps of 20 K. Table 1 contains descriptions of the systems studied.

**Table 1:** A brief description of each system in our simulations

# of glucose in the chain	Temperature (K)	Simulation time (μs)	# of water molecules	Initial box dimension (nm)	Final box dimension (nm)
10	280	1	2779	5	4.91
10	300	1	2779	5	4.94
10	320	1	2779	5	4.95
10	340	1	2779	5	4.99
10	360	1	2779	5	5.03
20	300	2	30 513	11	10.88
30	300	1.5	103 617	16.5	16.34

We use the GROMACS 2016.4 package^38^ to run our simulations. For the forcefield, we employ the CHARMM36 force field, developed by MacKerell group,^39^ which has shown promising results in simulating carbohydrate systems.^40^ In each simulation, a single chain of amylose is centred in a dodecahedron box and the system is solvated with TIP3P^41^ water molecules (Table 1), which is the same water model used in developing carbohydrate parameters in the CHARMM36 forcefield.^39^ The box size is chosen to prevent the chain from interacting with its mirror images in all periodic boundary conditions. After solvating the box, the system undergoes an energy minimization step to correct the position of any misplaced particles in the simulation box (*e.g.*, overlapping water molecules). In the next step, we run simulations to equilibrate the system under NVT condition for 2 ns with a time step of 1 fs. The position of atoms in the amylose chain (except for the hydrogens) is restrained during this NVT simulation to prevent any structural changes during this step. However, water molecules can move freely within the box. Following this step, the position restraint is removed and the time step for the simulation is set to 2 fs. In the following “production run”, the system is simulated under NPT conditions at 1 atm, using Parrinello-Rahman isotropic pressure coupling, with *τ*_p_ = 5 ps and compressibility = 4.5 × 10^−5^ bar^−1^.

For amylose chain of size 10, simulations of 1 μs are conducted across a range of temperatures. At *T* = 300 K, chains of size 20 and 30 are also simulated for 2 μs and 1.5 μs respectively. GROMACS tools and the VMD 1.9.3 (ref. 42 and 43) hydrogen bond plug-in, as well as custom C++ and python codes, are used to analyze the results of the simulations. VMD 1.9.3 is used to visualize our system.

### 2.2 Definition of imperfect-helical structures

**Fig. 2:**
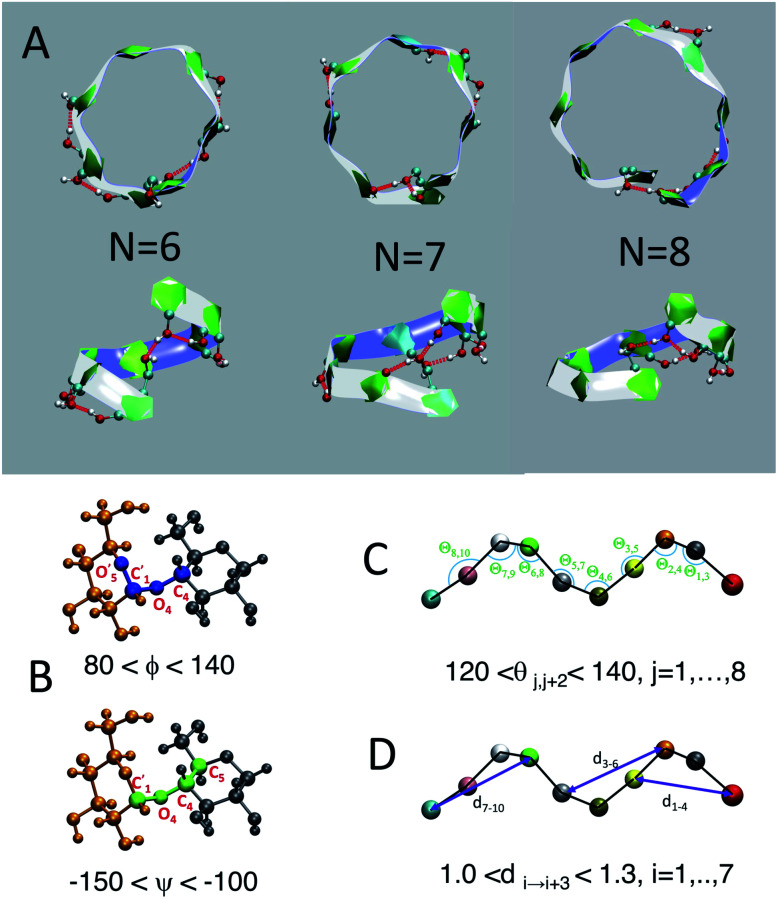
Perfect helix geometry: (A) top and side views of typical H-bonded full-turn helices captured in the *N* = 30 system; (B) torsion angles *ϕ* (in blue) and *ψ* (in green); (C) angle *θ*; (D) distance *d*. In A, each ball represent an atom in a glucose molecule. In C and D each ball represent the center of mass of each glucose unit.

We employ a geometric approach to capture imperfect-helices on amylose chains in the MD simulations. Based on our definition, imperfect-helices have similar geometries to the perfectly H-bonded helices, without the need for the precise H-bond patterns. To build our algorithm, we first search for H-bonded helices of size 6, 7 and 8 in our simulations and then use these as the template structures to extract their geometrical properties (Fig. 2-A). Since perfectly H-bonded helices do not appear in the amylose chain simulations in water, a less rigid version of the H-bond patterns is used to find these helical structures. Further details on this process are provided in ESI Section 2.†

Next, the properties of these template helices are used to develop three criteria to capture imperfect-helical geometries in the simulations: (1) torsion angles *ϕ* and *ψ*, (2) angle *θ* and (3) distance *d* (Fig. 2-B–D).

(1) *ϕ* (∠C4–O4–C′1–O′5) and the *ψ* (∠C5–C4–O4–C′1) are torsion angles formed by three consecutive bonds connecting the adjacent glucose units (Fig. 2-B). Based on our calculations, for an imperfect-helix the *ϕ* values should be between 80° and 140° and the *ψ* values should be between −100° and −150°.

(2) *θ* is the angle between the center of masses of three consecutive glucose residues (*θ*_*j*,*j*+2_) (Fig. 2-C). The *θ* value is calculated to be in the range of 120° to 140°, for an imperfect-helical structure.

(3) *d* represents the distance criterion in our method. Although a full-turn helical configuration has a length of at least 6 glucose units, we set the smallest imperfect-helical structures to have a size of 4 to capture partial helices in our structures. Thus, we use the distance between the center of masses of the *i* and *i* + 3 glucose units, where 1.0 nm < *d* < 1.3 nm (Fig. 2-D).

The ranges for these three criteria are chosen wide enough to capture the imperfect-helices that are geometrically similar to any of the H-bonded helices of size 6, 7 and 8. In our definition of the helical geometry, the torsion angles *ϕ* and *ψ* define a short-range arrangement between two consecutive glucose units in an amylose chain. Using only the torsion angles would result in capturing random structures, as discussed in the (ESI Section 3†).

In addition to this measure of local arrangement, longer range metrics are needed to capture imperfect-helices in simulations. Here, we use angle *θ* and distance *d* values, which reflect the arrangement between three and four consecutive glucose units in the chain respectively. Using the combination of torsion angles with only the angle *θ* (without the *d*) or only the distance *d* (without the *θ*) will still lead to capturing random non-helical structures (ESI Section 3†). Although adding the *θ* and the *d* will sacrifice some imperfect-helical structures, it will guarantee the absence of random structures in our results.

The range of values obtained for the torsion angles and the *θ* angles are in agreement with experimental results for the amylose chains in helical structures.^12,44–48^ To have the smallest imperfect-helical structure containing 4 glucoses, 6 torsion angles, 2 consecutive angles and one distance criteria must be satisfied (Fig. 2). Note that our approach allows us to determine whether an individual glucose is part of a helix-like conformation. More details on how to interpret these values to helices can be found in the (ESI Section 4†). Further, the range of parameters is chosen to be conservative and errs on the side of excluding imperfect-helices rather than admitting random structures (ESI Section 4†).

## 3 Results and discussions

### 3.1 System evolution

**Fig. 3:**
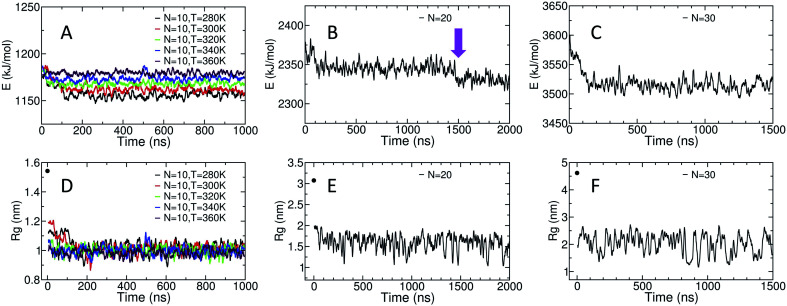
(A)–(C) Display the time evolution of the potential energy of the dihedral angles and (D)–(F) show the time evolution of the radius of gyration. The black data point at *t* = 0 ns in D, E and F indicates the initial *R*_g_ value for each system. All displayed data is calculated *via* running averages over 1000 data points, each 10 ps apart.

The potential energy of the dihedral angles and the radius of gyration (*R*_g_) of the chain generally decrease within the first 200 ns of the simulation (Fig. 3). These results indicate that the effect of the initial structure on the results disappears within the first 200 ns of the simulations.

For the amylose chain of length 10 (*N* = 10), the energy and the *R*_g_ plot are essentially constant after 200 ns. However, for systems at higher temperatures (*i.e.*, *N* = 10, *T* = 340 K and *N* = 10, *T* = 360 K), the decrease in the potential energy is not very significant and the system relaxes more quickly. Never-the-less, we consider the first 400 ns of the simulation as the relaxation time for the system. Thus, the last 600 ns of the simulation is used to study different properties of the amylose chain of size 10, unless stated otherwise.

Fig. 3-B shows the potential energy of the dihedrals for the *N* = 20 system at *T* = 300 K. In addition to the changes in the energy in the first ∼200 ns there is a distinct step down to a new lower energy value at *t* ∼ 1.5 μs (indicated by the magenta arrow). This sudden decrease likely indicates the chain overcomes a metastable structure. Thus, we use the last 500 ns of the system to calculate our results, unless stated otherwise. Results concerning the origin of the drop in energy at *t* ∼ 1.5 μs will be discussed in more details further in the paper.

For the amylose chain of length 30, the energy value does not change dramatically after the first ∼200 ns (Fig. 3-C). Here, we consider the first 500 ns as the relaxation time and use the last 1 μs of the simulation to study the behaviour of the system, unless stated otherwise. Note that for both *N* = 20 and *N* = 30, while there are significant fluctuations in *R*_g_, the (locally) mean value remains relatively constant (Fig. 3-E and F).

### 3.2 Imperfect-helical structures

**Fig. 4:**
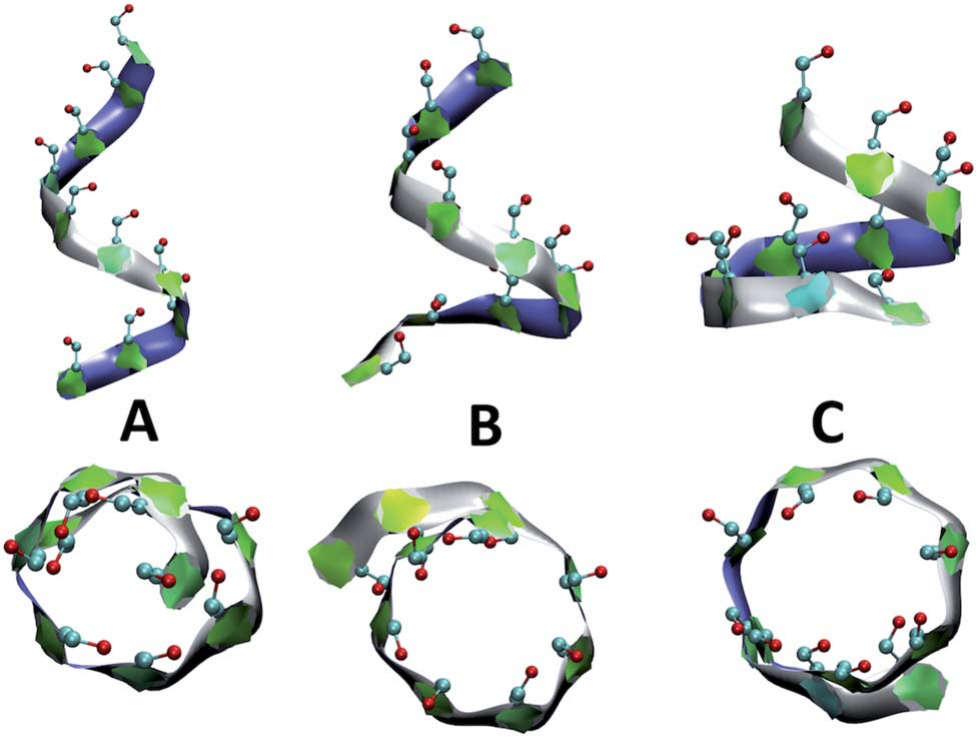
Side and top views of selected full-turn imperfect-helical structures for *N* = 10 at *T* = 300 K: (A) imperfect-helical structure containing properties of a helix of size 6; (B) structure containing properties of a helix of size 6 in the upper part and properties of a helix of size 7 at the lower part; (C) structure containing properties of a helix of size 8. The C5–C6–O6 atoms are shown explicitly.

Fig. 4 presents snapshots of full imperfect-helical structures captured by our algorithm for *N* = 10 at *T* = 300 K. In these structures, all 10 glucose monomers in the chain (and the bonds between them) satisfy the criteria outlined for imperfect-helices. Since our four criteria of imperfect-helicity (*ϕ*, *ψ*, *θ* and *d*) are obtained from properties of helices of size 6, 7, and 8, the structures captured by our approach will in general exhibit a combination of features from these different base helices (Fig. 4). For example, in Fig. 4-A, the clearly helical structure is tapered and the diameter of the helix decreases along the chain. The criteria that we have developed thus succeeds in capturing these “helical but imperfect” structures that cannot be identified in other studies based on H-bond patterns only.

**Fig. 5:**
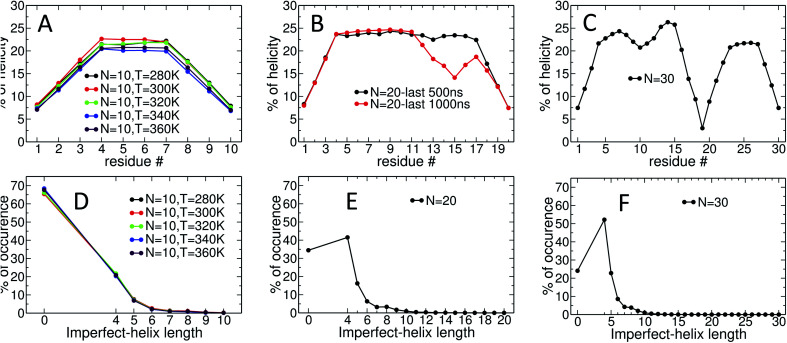
Time-averaged imperfect-helicity per residue for: (A) *N* = 10 at different temperatures over the last 600 ns of simulation; (B) *N* = 20 over the last 500 and 1000 ns of the simulation; (C) *N* = 30 over the last 1000 ns of the simulation. The probability of finding at least one helix of a particular size for: (D) *N* = 10 at different temperatures over the last 600 ns of simulation; (E) *N* = 20 over the last 500 ns of the simulation; (F) *N* = 30 over the last 1000 ns of the simulation. In (D, E and F), if more than one imperfect-helices of the same size occur in the chain at the same time, only one of them is counted. If imperfect-helical structures of different sizes occur in the chain at the same time, each one is individually counted.

Fig. 5 provides detailed information on the distribution of imperfect-helices for the different polymer lengths and simulation conditions. The results show that the first three residues on each end of the amylose chain have a lower probability of being found in an imperfect-helix state (Fig. 5-A–C). This finding is mainly an artifact of how we calculate the imperfect-helical regions in our algorithm. In brief, the first glucose on each end of the chain can only be part of a helix that includes the next three units. The second glucose on each end of the chain can be a part of two different helices (*e.g.*, glucose 1 to 4 or glucose 2 to 5) and so on. This increasing multiplicity yields an increasing probability until glucose 4 and higher (on each end of the chain) where the maximum number of helices is 4 (details are explained in the ESI Section 4†). In addition, the higher structural freedom in both ends could play a role in decreasing the amount of imperfect-helicity in these regions.

Residues near the centre of the *N* = 10 chain (*i.e.*, glucose-4 to glucose-7) have similar probabilities of being found in an imperfect-helix with an average value around 22% (Fig. 5-A). These results show a very weak temperature dependency and no clear ordering in the mean probability as a function of temperature.

For the *N* = 20 chain, the results vary significantly with the portion of the trajectory that is analyzed. In the last 500 ns of the simulation, the curve is similar to that for the length 10 systems; excluding the first and last three, residues have average imperfect-helicity values around 23% (Fig. 5-B). However, in the last 1 μs of the simulation, there is a region with very low imperfect-helicity centred on residue 15 and spanning residues 13–17. Recall that, as shown in Fig. 3-B, the system overcomes a metastable configuration yielding a drop in potential energy at *t* ∼ 1.5 μs, *i.e.*, within the last 1 μs of the simulation. These results suggest a link between the low imperfect-helical region and the metastable configuration in this system.

For the *N* = 30 chain, there is a sequence of glucose units with low imperfect-helicity probabilities centred on residue 19 (including residues 17, 18, 19, 20 and 21) (Fig. 5-C). This region is similar to the low imperfect-helical region in the amylose chain of size 20 in the last 1 μs. However, unlike the chain of size 20, there is no dramatic change in the potential energy of the amylose chain of length 30 beyond the first 200 ns of the simulation (Fig. 3-B and C). Thus, this low imperfect-helicity region suggests that the *N* = 30 amylose chain is in a metastable configuration that is not escaped during the simulation. Despite this non-helical region on the chain, the rest of the chain has imperfect-helical probabilities ranging from ∼20–25% with an average around 22% excluding the first and last three residues (consistent with results above).

The amylose chains of length 10, 20 and 30 at *T* = 300 K show similar averaged imperfect-helicity probabilities around ∼22% (excluding the first and last 3 residues in all systems as well as residues 17, 18, 19, 20 and 21 in the system of size 30). Thus, there is a weak dependence of the probability of forming imperfect-helices and the length of the amylose chain.

Analyses of the distribution of imperfect-helical regions show that in ∼65–70% of the simulation time, the *N* = 10 amylose chains do not contain any imperfect-helices (Fig. 5-D). Note that these curves have a very weak dependence on temperature. This probability decreases to ∼35% and ∼25% as the length of the amylose chain increases to 20 and 30 glucose units respectively (Fig. 5-E and F). Correspondingly, an imperfect-helix of at least length 4 is found 65% of the time at *N* = 20 and 75% of the time at *N* = 30.

Unsurprisingly, the shortest possible imperfect-helix of length 4 has the highest probability of occurrence. This probability increases from ∼20% for *N* = 10 to ∼41% for *N* = 20, up to ∼52% for *N* = 30. The probability of finding longer imperfect-helical regions decays rapidly with the length of the helix. For example, in a chain of size 10, the probability of finding an imperfect-helix of length 5 is ∼7% and is ∼2% for length of 6 (Fig. 5-D).

As the length of the amylose chain increases, finding shorter individual imperfect-helical regions at different parts of the chain at the same time becomes more likely. In Fig. 5-D–F, if multiple imperfect-helices occur on the chain at the same time, they are counted independently. However, if they are of the same size, only one of them is counted. For example, if three helical regions of size 4, 4 and 5 occur on the chain at the same time, these helices will be counted as one imperfect-helix of size 4 and one imperfect-helix of size 5 in Fig. 5-D–F. The total helicity of the chain at any time can also be calculated (details are explained in the ESI Section 5†).

**Fig. 6:**
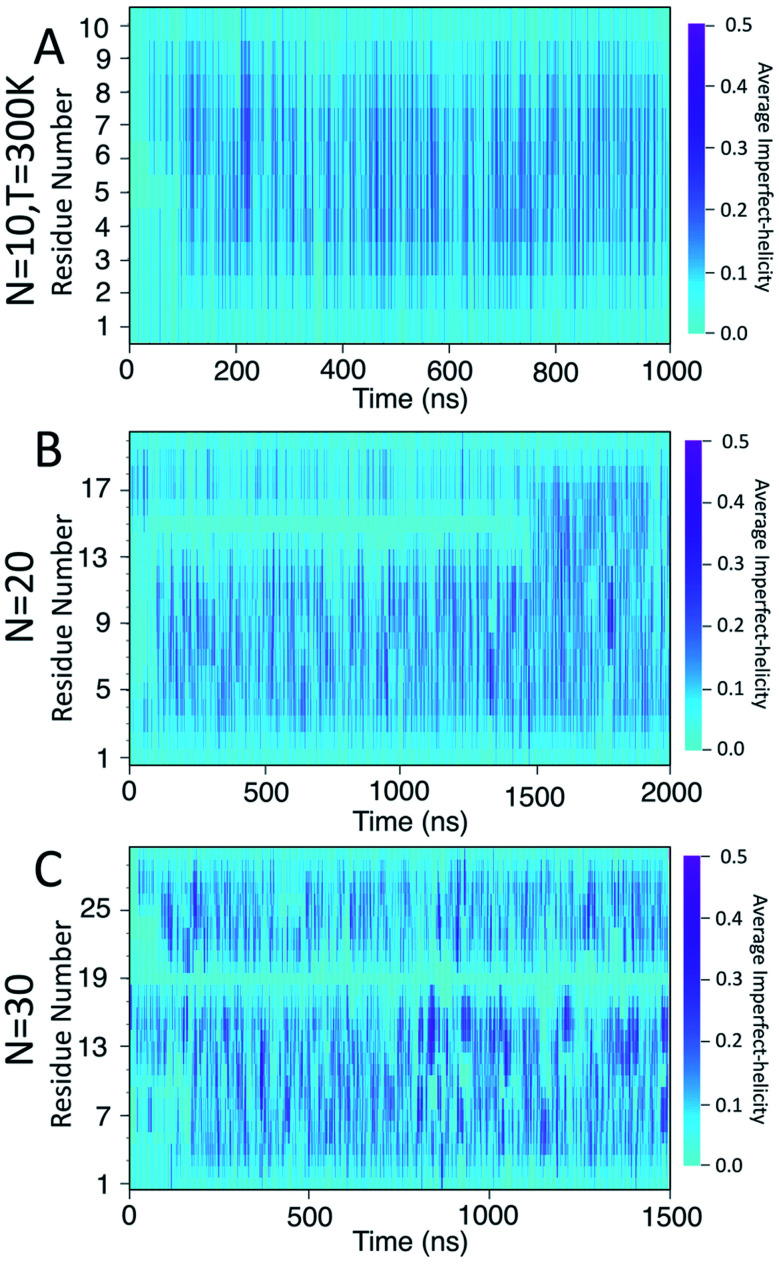
Heat map of the average likelihood of imperfect-helical structures for: (A) *N* = 10; (B) *N* = 20; (C) *N* = 30. Each data point is averaged over 100 frames, each 10 ps apart. The light cyan colour represents non-helical regions (average helicity of 0). The darker cyan regions (and brighter magenta regions) indicate the average amount of helicity found, with magenta corresponding to 50% helicity.

To examine the dynamics of imperfect-helix formation, we begin with 2D heat map plots of the helicity as a function of time (Fig. 6). The data is averaged over a time window of 1 ns to produce a number between 0 and 1 where 0 corresponds to that residue never being in a helical state during that 1 ns while 1 corresponds to it being in a helical state for the entire trajectory. In the figure, light cyan correspond to 0, magenta to 0.5, and the shade of darker colours to a number in between the two. Magenta thus corresponds to units that are found to be helical 50% of the time; all helicity values are lower than this value.

In all systems, the heat map is lighter in the first few 100 ns of the simulation which arises from initializing the chain with no imperfect-helix sections. This is in agreement with the dihedral potential energy of the chain in the first few 100 ns (Fig. 3). Moreover, during the simulation, the first three residues on each end of the chain are lighter than other residues – again indicating low probability of being in a helical state and in agreement with Fig. 5-A–C.

For the chain of length 10, at *T* = 300 K, no dramatically magenta or light cyan regions appear at any particular time (Fig. 6-A). However, for *N* = 20, the chain has a long-lasting region with very low probability of being helical that is centred on residue 15 for the first ∼1.5 μs of the simulation (Fig. 6-B). This non-helical region is strongly correlated with the jump in the dihedral potential energy at *t* ∼ 1.5 μs (Fig. 6-B and 3-B). When the persistent low helicity probability region disappears from the heat-map plot, the dihedral potential energy decreases to a lower value, which could indicate overcoming a metastable structure. Similarly, for *N* = 30, the heat map has a long-lasting low helicity probability region centred on residue 19 (Fig. 5-C). As opposed to the *N* = 20 data, this region persists for the entire length of the simulation. Due to the large number of particles in this system (∼3 times more particles than *N* = 20 and ∼37 times more particles than *N* = 10 (Table 1)), it was not possible to run simulations long enough to see if this persistent region would eventually disappear.

### 3.3 Dynamics of imperfect-helices

The heat maps shown in Fig. 6 demonstrate that the helical sections of the chain are quite dynamic and there are both long- and short-time variations of the amount of helicity in different parts of the chain. In this section, the dynamics are explored by examining sources of the fluctuations and also the long lasting non-helical regions.

#### 3.3.1 Band-flips

**Fig. 7:**
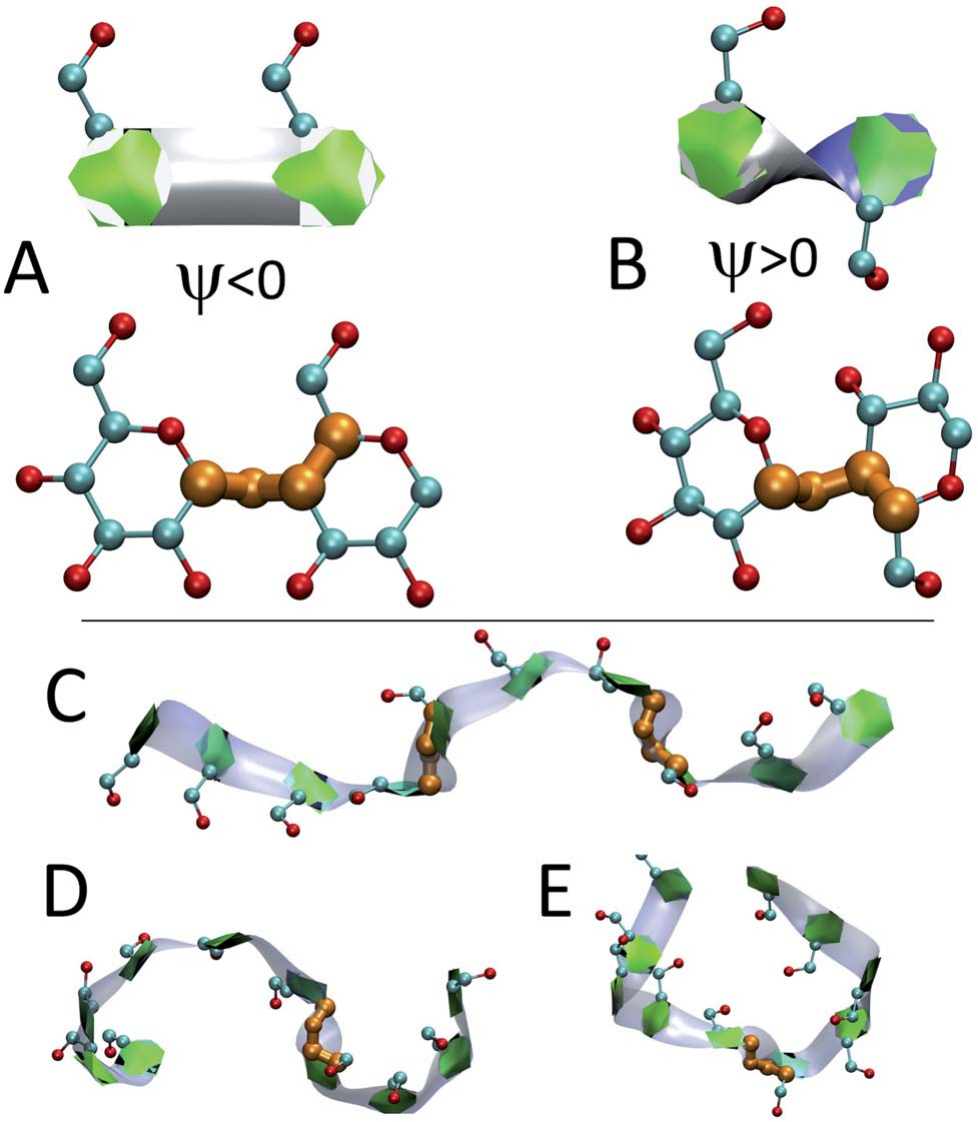
Band-flipped structures and their effect on the structure of amylose chains: (A) two consecutive glucose units in extended structure where *ψ* is negative and shown in orange; (B) two consecutive glucose units in band-flipped structure where *ψ* is positive; (C) extended conformation of an *N* = 10 chain induced by the presence of two band-flips on bonds 3 and 7; (D) two imperfect-helical structures of sizes 4 and 6 separated by a band-flip bond 6. (E) Two imperfect-helical structures of size 5 separated by a band-flip on bond 5.

A source of fluctuations in the helicity measurements is “band-flips”. Band-flips correspond to the *ψ* dihedral angle changing significantly such that the orientation of two glucose units changes from parallel (*ψ* < 0) to antiparallel (*ψ* > 0) (Fig. 7).^15,21,45,49^ Giving that this entails a large change in a dihedral angle, band-flips are intrinsically helix breakers in our approach.

In addition to modifying local structure, band-flips can affect the whole amylose chain by inducing extended conformations (Fig. 7-C) or breaking a long imperfect-helical structure into two (Fig. 7-D and E). However, while a band-flip breaks a helix, overcoming a band-flip configuration does not necessarily produce imperfect-helical structures.

**Fig. 8:**
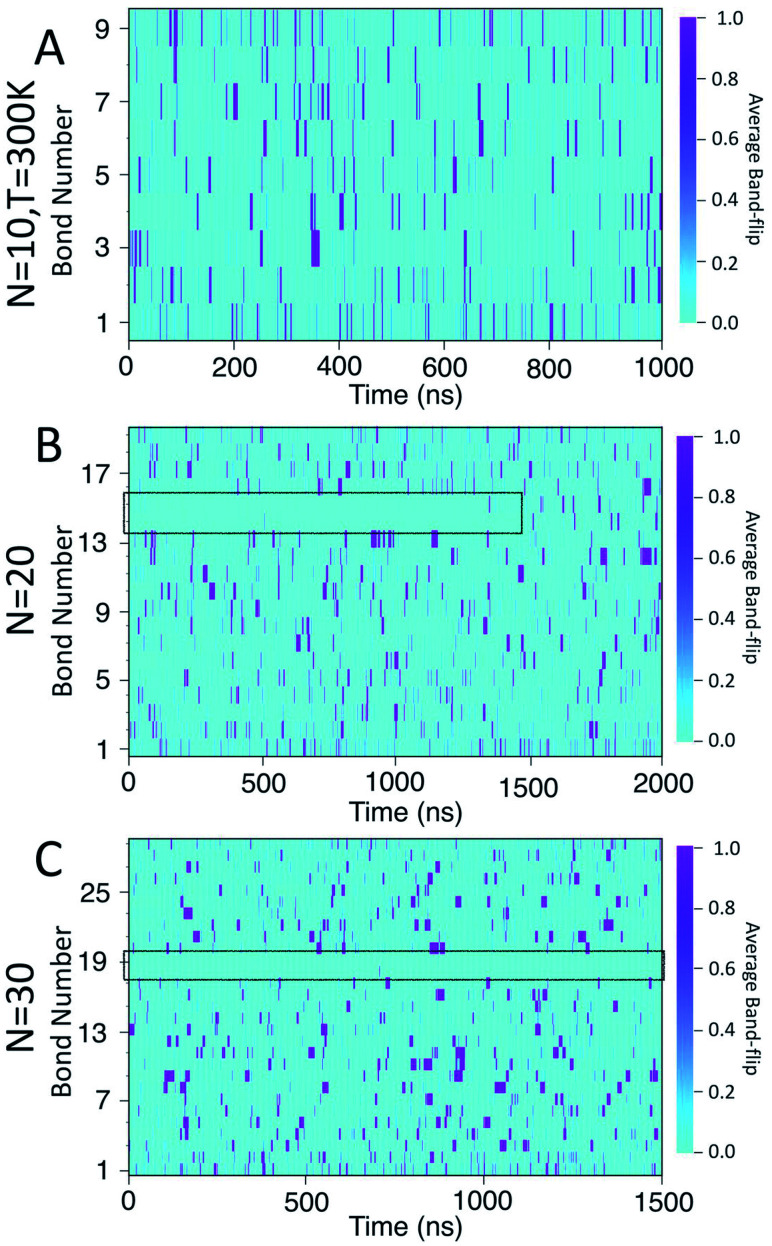
Heat maps of the average likelihood of finding band-flipped bonds for: (A) *N* = 10; (B) *N* = 20; (C) *N* = 30. The colour scale ranges from light cyan, indicating no band-flips found, to magenta which indicates that a band-flip is present for the entire time window.

Fig. 8 presents a 2D heat-map of the averaged band-flipped configurations on each bond over 1 ns time frames in the simulations. The light cyan colour represents non-band-flipped bonds and magenta indicates band-flipped bonds. In all of the simulations, the band-flips occurring on the first or the last bond of the chain have a shorter lifetime, *i.e.*, narrow magenta lines. However, band-flips that occur on the rest of the chain can have longer lifetimes, *i.e.*, thick magenta lines. The band-flips on the edges of the chain flip back by rotating only one glucose unit, while for the ones in the middle, several glucose units need to rearrange.

In the case of the *N* = 10 amylose chain, band-flips are present on all bonds during the simulation. For *N* = 20, for the first ∼1.5 μs of the simulation, band-flips rarely appear on bonds 14 and 15, which connect residue 15 to its neighbouring residues. This region and time corresponds to the long-lasting non-helical section in Fig. 6-B and the drop in dihedral potential energy (Fig. 3-B). Similarly, for *N* = 30, the band-flipped structures rarely occur on bonds 18 and 19, which connect residue 19 to its adjacent residues on the chain – in agreement with very low imperfect-helicity patterns over residue 19 (Fig. 5-C and 6-C). While the band-flip behaviour is correlated to the long-lasting behaviour seen in other measurements, it is important to note that the bonds in question are not band-flipped and thus cannot be the long-lasting helix breaking mechanism. This is further cemented by noting that while the time scale of band-flip fluctuations is significant, it is still much smaller than the long-lasting events and thus band-flips are unlikely to be the cause of these metastable structures.

#### 3.3.2 Turn-like structures

**Fig. 9:**
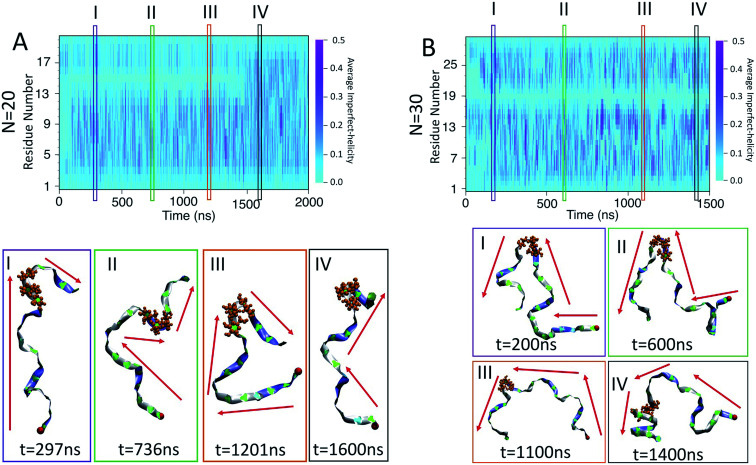
Kink structures in the amylose chains: (A) heat map of helicity for *N* = 20; (B) heat map of helicity for *N* = 30. Snapshots corresponding to selected time frames I–IV are included below the heat maps. Red arrows indicate the general orientation of the amylose chain and the red bead represents the first residue of the chain. The orange residues in CPK representation show residues 14, 15 and 16 for *N* = 20 and residues 18, 19 and 20 for *N* = 30.

The time scale of the local fluctuations described above indicates larger scale rearrangements in the chain configuration may be responsible for the apparent metastabilities observed in the *N* = 20 and *N* = 30 systems. Snapshots of the *N* = 20 and *N* = 30 amylose chains show the presence of multiple secondary structures on the chain including random coils, band-flips, and imperfect-helices at the same time (Fig. 9). Furthermore, sudden changes in the direction of the chain arising from “kinks” are apparent.

For *N* = 20, there is a persistent kink that is observed for selected frames I, II, and III (Fig. 9-A). This kink yields a sudden change in the direction of the chain (indicated by red arrows) which, by our criteria, means that no helix can form in this area. This then is the cause of the long lasting light cyan space in the average helicity heat maps (Fig. 6-B) and the band flips plot (Fig. 8-B). In frame IV of Fig. 9-B, selected soon after 1500 ns, the kink is resolved and the chain a more consistent orientation. This overcoming of the kink also yields the drop in dihedral potential energy seen in Fig. 3-B and explains the difference of the helicity propensity for individual residues between the first part and second part of the simulation (Fig. 5-B).

Similar results are found for the *N* = 30 case (Fig. 9-B). Here, however, the kink is not resolved and is apparent in all frames. The sudden change in the chain orientation introduced by the kink is a helix breaker for the entire simulation – even when the chain beyond the kink adopts a clear imperfect-helix structure as shown in selected frame IV.

While these kinks can arise anywhere, it is interesting that once they form in one place they do not seem to travel along the chain. For *N* = 20 the location is consistently around residue 15 and for *N* = 30 it is consistently around residue 19. Further, for *N* = 20, resolution of the kink happens rapidly as the light cyan space at residue 15 suddenly disappears (Fig. 6-B). Hence, the kinks do not seem to be local rearrangements (as opposed to band-flips) but instead allow for larger scale rearrangements of the sub-chains on either side of the kink. Resolution of these metastabilities thus also requires large scale rearrangements and hence long time scales.

These kinks in the amylose chain are thus acting similarly to turn regions in the secondary structures of proteins.^50^ They help to define the limits of other secondary structures (imperfect-helices here, α-helices and β-sheet regions in proteins) and give a high degree of flexibility allowing for larger scale, orientational rearrangements of the secondary structure on either side.

To verify the efficacy of the geometric criteria introduced herein, limited simulations were performed for the amylose chain of size 10, at *T* = 300 K, using the TIP4P^51^ water model. The details of the results are somewhat different – largely due to finding a kinked structure in the TIP4P system and not in the TIP3P system (see ESI Section 1†). It is unclear if this is simply a statistical difference or more directly the result of the water model. Regardless, the TIP3P results are more reliable as that is the water model that was used to develop the carbohydrate potential we employed. Further, analysis of both sets of results confirm the accuracy and robustness of the proposed geometric criteria in terms of (i) identifying helical sections, (ii) mapping the dynamics of these sections to molecular details such as band-flips and (iii) identifying kinks that have significant impact on the conformations of the chain.

## 4 Conclusion

In this paper, the structure and dynamics of single amylose chains of different lengths at various temperatures were studied using MD simulations (Table 1). A set of geometry-based criteria was developed as a means of identifying parts of the chain that are found in helical conformations. These criteria allow for the detection of conformations that are geometrically helical even if they do not satisfy stricter definitions of helicity based on H-bond patterns. However, by still being local rather global criteria, they also allow for discrimination of helicity/non-helicity for different regions of the chain. In our scheme, the shortest possible helix is of length 4 while the longest is the entire chain being helical. We call these conformations “imperfect-helices” in contrast to the strict, H-bond defined “perfect” helices.

Using these criteria, it was shown that amylose chains of all lengths and all temperatures contained a significant degree of imperfect helicity. At *T* = 300 K, at least one helix with a length of at least 4 is found ∼35% of the time for *N* = 10, ∼65% for *N* = 20, and ∼75% for *N* = 30. Hence, although they do not exhibit precise H-bond patterns, single amylose chains in water do possess a large degree of geometric helicity. Although helices of length 4 are the most common, longer helices are also found frequently. These results demonstrate that helicity is a major secondary structure conformation for single amylose chains in water. This is in contrast with studies that use H-bond patterns to define helices, but that is by design.

Helicity along the chain was shown to be very dynamic. While the longer chains usually have some degree of helicity, the helices form and dissolve on the order of 10–100 s of nanoseconds. Thus, helix formation and stability in amylose are much more fluid than α-helices in polypeptides.

The findings of significant helicity that is very dynamic in nature are in agreement with the experimental and simulation studies that conclude that amylose chains in water contain random sections of helicity that rapidly transfer between helical and coiled states.^22–27^ Our methodology generates this validation in the absence of strict H-bonding criteria but maintains determination of helicity at a single glucose scale such that the dynamics are well resolved.

Examination of the dynamics also revealed the existence of helix breakers. Band-flips in the dihedral angles prevent helix formation for time scales on the order of 1–10 ns. Much longer periods of extremely low helicity are also observed for the longer chains. There is a section of the *N* = 20 chain that is never helical for the first 1.5 μs of the simulation and a section of the *N* = 30 chain that is never helical for the entire 1.5 μs simulation. These long-lasting non-helical sections were shown to be due to “kinks” that cause sudden rearrangement in the orientation of the amylose chain. Similar to turn sections in polypeptides, these kinks allow for a high degree of flexibility permitting arrangement of the subchains on either side of the kink – which typically exhibit a degree of helicity. The kinks were shown to be stationary in that they did not travel up and down the chain but remained centred on one glucose monomer. For the *N* = 20 case, the resolution of the kink also happened quickly, which again indicating that the kink did not travel to an end of the chain and then disappear. Instead, the kink was resolved by large scale rearrangement of the subchains that restored the overall orientation of the amylose chain. Resolution of the kink was correlated to features in many other measurements including a drop in the total dihedral energy of the chain, normalization of the helix propensity measurements, and termination of the suppression of band-flips.

Enabled by the geometric criteria introduced herein, this study has established that helical conformations are a key secondary structure for amylose chains in water. Further, kinks in the chains can allow for metastable, tertiary arrangements of subchains. These fundamental structural aspects are both critical for understanding low-density solutions of amylose and serve as a starting point for making sense of denser – and more complicated – amylose-chain-containing structures such as glycogen nanoparticles.

Further, the success of the methodology introduced in this paper for characterizing the structure and dynamics of single amylose chains is encouraging in terms of using this approach in other systems. The geometric approach we outline could easily be adapted to characterize the structure and dynamics of various polymers, including different carbohydrates and polypeptides. Applications could include studying the random coil sections of folded proteins or to develop looser criteria for identifying secondary structure like regions that contain a relatively high fluidity of hydrogen bonding, including intrinsically disordered proteins.^52^

## Conflicts of interest

There are no conflicts to declare.

## Supplementary Material

RA-011-D1RA00071C-s001
