# Supplementary material for: Using geometric criteria to study helix-like structures produced in molecular dynamics simulations of single amylose chains in water
Source: RSC Adv. 2021 Mar 24;11(20):11992–2002. doi: 10.1039/d1ra00071c (PMC8697119; doi:10.1039/d1ra00071c)
Supplement: RA-011-D1RA00071C-s001 [file RA-011-D1RA00071C-s001.pdf]

Electronic Supplementary Material (ESI) for RSC Advances.
This journal is © The Royal Society of Chemistry 2021

Supplementary Data for Using Geometric Criteria to Study Helix-like Structures Produced in Molecular Dynamics Simulations of Single Amylose Chains in Water

Mohammad Hassan Khatami, William Barber, and Hendrick W. de Haan*

Ontario Tech University, Department of Physics, 2000 Simcoe St N
Oshawa, ON, Canada L1H 7K4

E-mail: Hendrick.deHaan@ontariotechu.ca

1 Comparing Results for the TIP3P and TIP4P Water Models

In this section of the supplementary data, we compare MD simulation results of an amylose chain of size 10 at T=300 K, in different water models, i.e., TIP3P[1] and TIP4P,[2] simulated using the same method described in the main paper. These simulations are labelled based on their water model, TIP3P and TIP4P, in this section of the supplementary data. The details of calculating the results discussed here are explained in the main text of the paper and the text in the other sections of the supplementary data.

Figure S1 represents dihedral potential energy and R_g of the amylose chain for both TIP3P and TIP4P systems. As discussed in the main text of the paper (figure 3-A), the dihedral potential energy of the TIP3P system decreases within the first 200 ns of the simulation. However, unlike the TIP3P system, the dihedral potential energy in the TIP4P simulations does not decrease during the simulation and remains higher compared to the TIP3P system. This higher potential energy agrees with the metastable configurations in the TIP4P system, which is discussed later in this section.

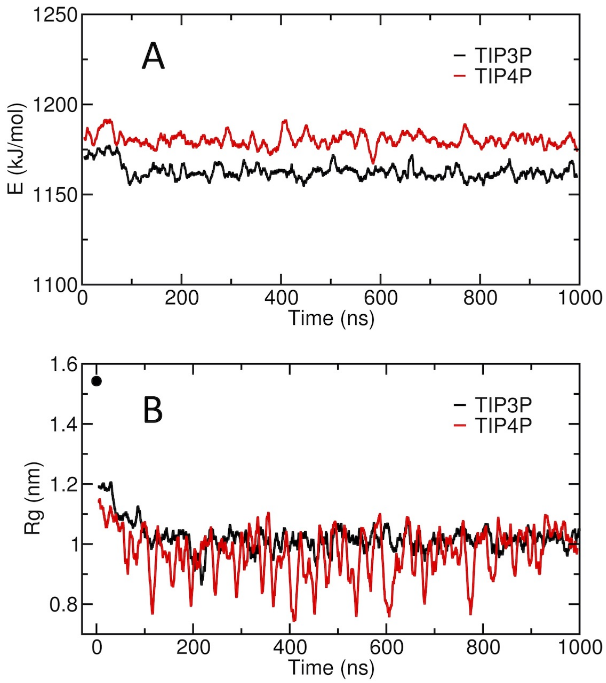

Figure S1: A) The time evolution of the potential energy of the dihedral angles and B) the time evolution of the radius of gyration for TIP3P and TIP4P systems. The black data point at $t = 0$ ns in B indicates the initial R_g value for each system. All displayed data is calculated via running averages over 1000 data points, each 10 ps apart.

Moreover, we can see that the R_g results for the TIP4P system have larger fluctuations compared to the TIP3P, which represents lower stability in the structure of the chain in the TIP4P

simulation (Figure S1-B).

Figure S2 represents the time-averaged distribution of imperfect-helices per each residue for the TIP3P and TIP4P systems. Unlike the

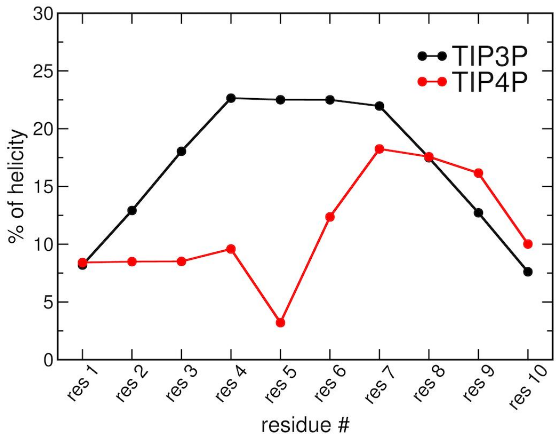

Figure S2: Time-averaged imperfect-helicity per residue for the TIP3P and TIP4P systems over the last 600 ns of the simulation.

TIP3P system, which has a uniform and symmetric pattern (i.e., increasing from one end, reaching a plateau and decreasing again), the pattern for the TIP4P system is nonuniform. It shows a region with very low imperfect-helicity centred on residue 5. This low imperfect-helicity pattern is similar to the findings for the $N = 20$ and $N = 30$ chains (Figure 5 A and B), suggesting the presence of long-lasting metastable configuration in the TIP4P system.

As the chain is only 10 residues long in the TIP4P system, the metastability on residue 5 (i.e. almost on the centre of the chain) changes the imperfect-helicity patterns of almost all residues. However, the first and last residue in the chain, which are located farthest from residue 5, has similar imperfect-helicity patterns to the TIP3P system. Additionally, we can see that the pattern of helicity is not symmetric around residue 5. This is mainly because the number of residues is not similar on each side, i.e., four residues on the left (residues 1, 2, 3, and 4) and five residues on the right (residues 6, 7, 8, 9 and 10). The effect of the number of residues in calculating the imperfect-helicity is discussed in the main paper and section 4 of the Supplementary data. In brief, helicity is determined using the state of four consecutive residues and thus there is a significant difference between a kink being 4 residues from one end and 5 from the other in terms of terms of reducing the helicity on either side. Noting this, the helicity for residues 7, 8, and 9 do compare well between water models. This indicates that the major difference is the presence of the kink rather than drastic changes in the helix form tendency for kink-less structures.

The 2D heat map plots of the average imperfect-helicity and band-flips for both TIP3P and TIP4P systems are provided in figure S3. The TIP4P system has a long-lasting non-helical region on residue 5, which remains for the entire simulation, which is in agreement with the results represented in figure S2 for the TIP4P system. This long-lasting non-helical region in the TIP4P system has a similar behaviour as the long-lasting non-helical regions for the $N = 20$ and $N = 30$ systems (Figure 6-B and C).

Similarly, the 2D heat-map of the averaged band-flipped configurations indicates a lack of band-flip configuration on bonds 4 and 5 in the TIP4P system (Figure S3-D). These two bonds connect residue 5 to its neighbour monomers, i.e., residues 4 and 6. The long-lasting lack of band flips on two consecutive bonds is similar to the results obtained in the $N = 20$ and $N = 30$ systems (Figure 8-B and C).

A general comparison between the results for the TIP4P system and the $N = 20$ and $N = 30$ simulations suggests the presence of kink configurations in the TIP4P simulation (Figures S3, 6 and 8). Figure S4 provides qualitative representations of kink configuration in the amylose structure in the TIP4P system. We can see that the chain forms different tertiary structures, similar to the findings for the $N = 20$ and the $N = 30$ systems (Figure 9).

The proposed geometric helicity criteria thus are successful at identifying imperfect helical structures and mapping this to helix forming propensity for individual glucose units along the chain for both water models. Further, kinks in the chain are identified and correlated to the cessation of band-flips and an increased potential energy in the dihedrals of the chain, in-

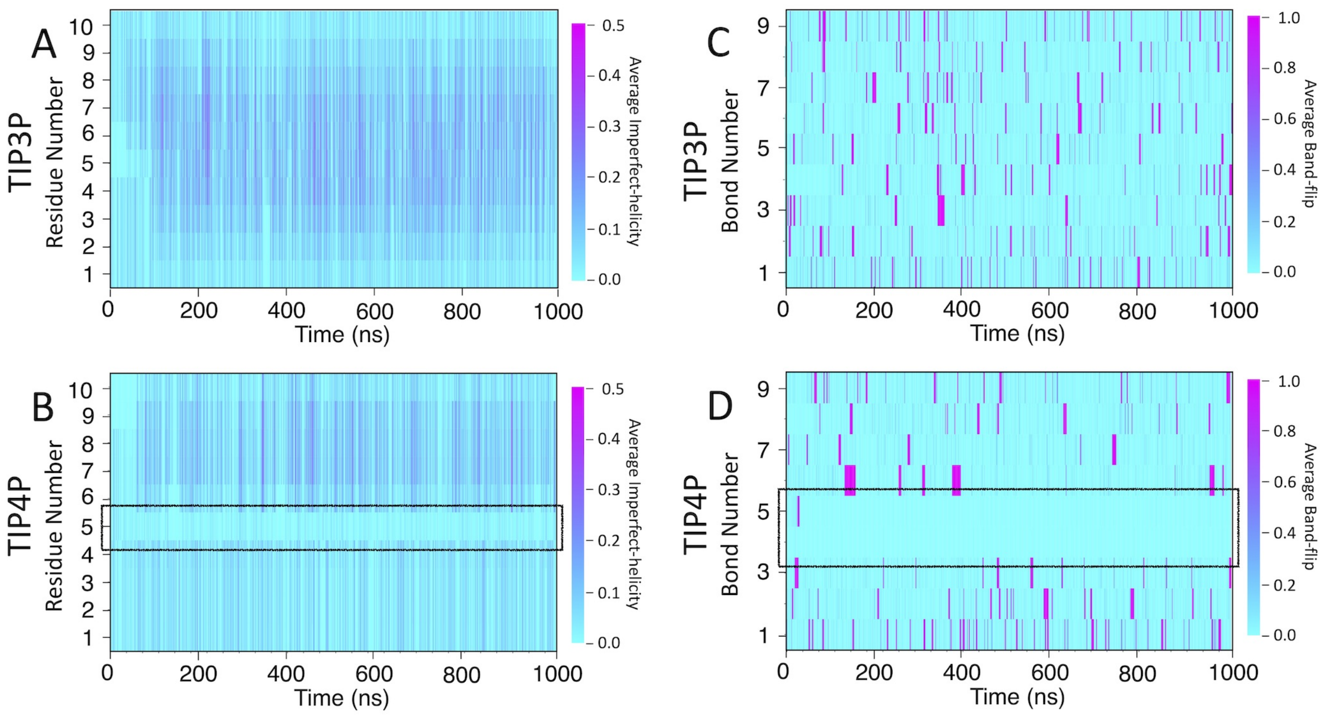

Figure S3: Heat map of finding the average likelihood of: A) imperfect-helical structures for the TIP3P system. B) imperfect-helical structures for the TIP4P system. C) finding band-flipped bonds for the TIP3P system. D) finding band-flipped bonds for the TIP4P system. Each data point is averaged over 100 frames, each 10 ps apart. The light cyan colour represents non-helical regions (average helicity of 0). For A) and B), the darker cyan regions (and brighter magenta regions) indicate the average amount of helicity found, with magenta corresponding to 50% helicity. For C) and D), the darker regions indicate the average amount of band-lips found with magenta corresponding to 1.

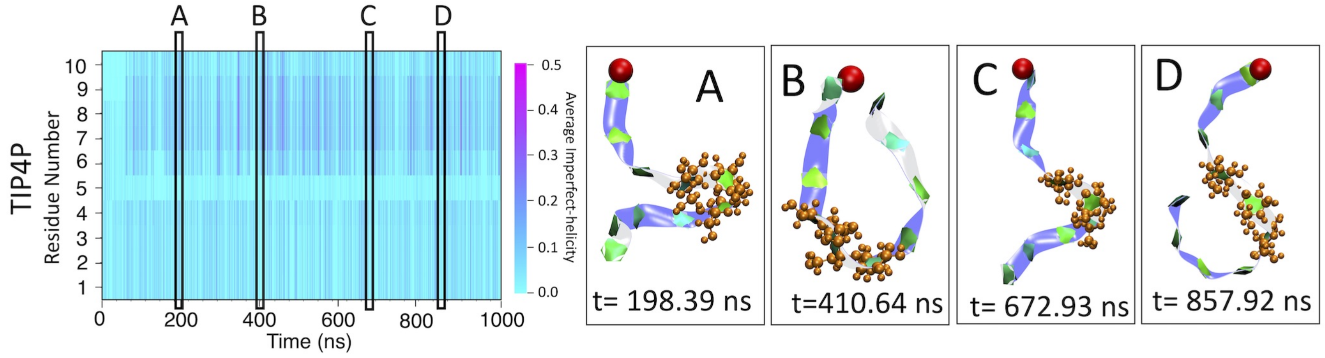

Figure S4: Kink structures in the amylose chain of the TIP4P system. Snapshots corresponding to selected time frames A–D are included on the right of the heat map. The red bead represents the first residue of the chain. The orange residues in CPK representation show residues 4, 5 and 6 of the amylose chain.

dicating a metastable state. These measurements and conclusions are consistent between the $N = 10$ kink found in the TIP4P and the $N = 20$ and $N = 30$ cases for the TIP3P water model. The primary difference is thus that a kink is found for the $N = 10$ chain in the TIP4P water model, but not for the $N = 10$ chain with the TIP3P water model. Further investigation

is required to explore whether this is simply a statistical effect or more directly caused by the difference in water models.

2 Finding Template Helices in MD Simulations

To find the geometrical criteria to capture the imperfect-helices in the amylose chains, we first search for template structures of H-bonded helices in the simulations. Since the amylose chains do not produce the perfectly H-bonded helices in water, less rigid conditions are used to find the templates in the simulation. The template structures should satisfy two main conditions. First, at least one intra-chain H-bond should occur between any glucose units of i and $i+6$ for a helix template of size 6, i and $i+7$ for a helix template of size 7, or i and $i+8$ for a helix template of size 8. Second, no H-bonds between non-consecutive residues in this region should be detected. For example, in the case of a template helix of size 6, O6 of residue 4 is H-bonded to O2 and/or O3 of residue 10. In addition, no H-bonds should occur between residues 5 and 7 or residues 7 and 10. If both conditions are satisfied, then the structure is considered as a template structure. At this point, there is no need for the H-bonds between O3 and O2 of the consecutive glucose units. The geometrical properties of these H-bonded templates are used to develop criteria to define and identify the imperfect-helices.

Some template structures captured in our simulations do not have circular symmetry, as shown in the top view in figure 2-A. The asymmetries in the templates occur because some of the H-bonds do not form in the chain as they should in a perfectly H-bonded helix. The asymmetrical structures could introduce values in the helicity criteria that do not correspond to a helical configuration. Thus, while defining the ranges for the ϕ, ψ, θ and the d, we ignore the top and the bottom ~10% of the values obtained from the template structures.

3 Capturing Random Structures

The ranges for the dihedral angles (ϕ and ψ), the angle θ and the distance d are chosen wide enough to capture imperfect-helices that are geometrically similar to H-bonded helices of size 6, 7 and 8. In our definition of the helical geometry, the torsion angles ϕ and ψ define a short-range arrangement between two consecutive glucose units in an amylose chain. Using only the torsion angles as our criteria would result in capturing random structures. As an example, figure S5-A shows a structure of size 8 that is captured based only on the torsion angles criteria, while the θ and d criteria are not satisfied.

Thus, we need longer range metrics to refine our criteria imperfect-helices. Here, we use the angle θ and distance d values, which represent the arrangements between three and four consecutive glucose units in the chain respectively. Using the combination of torsion angles with only the angle θ (without the d) or only the distance d (without the θ) will still lead to capturing random non-helical structures (Figure S5-B and C).

4 Capturing Imperfect-helices

In this section, the algorithm to capture imperfect-helical structures on the amylose chains is described. Since the shortest imperfect-helix contains 4 glucose units, the chain is divided into several sets of size 4. For example, in the amylose chain of size 10, the chain is divided into 7 sets, where set #1 represents residues 1 to 4, set #2 represents residues 2 to 5, ..., and set #7 represents residues 7 to 10 (Figure S6-A). If the four residues and the bonds between them satisfy the criteria, then the set is considered an imperfect helix: all residues are counted as being in a helical state and their helicity is set to 1. If this set does not meet all criteria, all monomers are considered non-helical and their helicity is set to 0 (Figure S6-B, C and D). If a residue is in an imperfect-helical configuration in at least

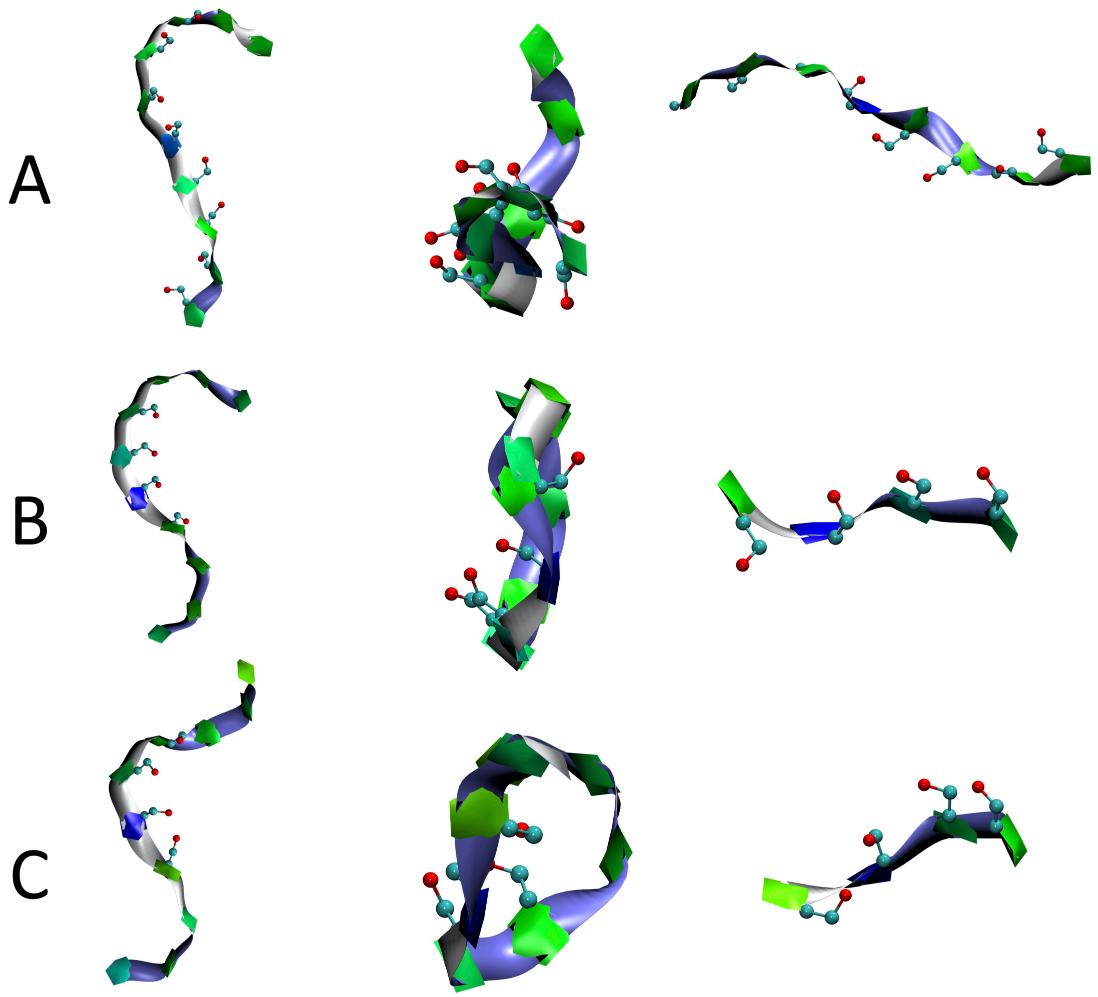

Figure S5: Snapshots of structures satisfying: A) only the dihedral angles ϕ and ψ criteria; B) only the dihedral angles ϕ and ψ and the angle θ criteria; C) only the dihedral angles ϕ and ψ and the distance d criteria. The left column presents a side view of the whole chain, the middle column presents the bottom view of the whole chain, and the right column presents an arbitrary view displaying the section of the chain that we are interested in it.

in one set, it is considered imperfect-helical residue, regardless of its assessment in other sets. For example, in case 1 in figure S6-B, sets #2, #3 and #6 are in non-helical configurations, but the whole structure is considered to be imperfect-helical.

In our algorithm, each residue is considered in four sets, except for the first 3 residues on each end. Figure S6 shows that the first, second and the third residues on each end of the chain are part of one, two and three sets respectively. Thus, the first 3 residues on the chain are not equally treated in our algorithm compared to other residues. As an example, if residue 5 is in three non-helical sets and one imperfect-helical set, it is considered helical. However, residue 1 (or equivalently residue 10) must always be in an imperfect-helical set to be considered helical.

It should be noted that, although more forgiving than the H-bond helix definition, our criteria for imperfect-helices are still quite conservative. For the shortest imperfect-helix (length of 4), 9 constraints, including 6 dihedral angles, 2 angles and 1 distance, need to be satisfied. Even if only one of the constraints is not satisfied, the structure is considered non-helical. Hence, short-time fluctuations – even from one frame to the next – can cause part of the chain to switch from helical to non-helical. Such rapid fluctuations are shown in Figure S7 where all the molecular structures look helix-like but the state of particular residues changes from frame

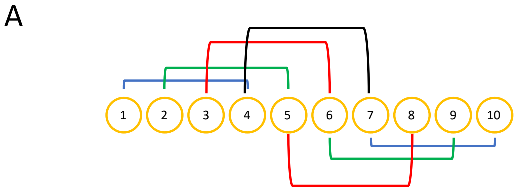

B

Case 1

	Res#1	Res#2	Res#3	Res#4	Res#5	Res#6	Res#7	Res#8	Res#9	Res#10
Set #1	1	1	1	1						
Set #2		0	0	0	0					
Set #3			0	0	0	0				
Set #4				1	1	1	1			
Set #5					1	1	1	1		
Set #6						0	0	0	0	
Set #7							1	1	1	1
TOT	1	1	1	1	1	1	1	1	1	1

C

Case 2

	Res#1	Res#2	Res#3	Res#4	Res#5	Res#6	Res#7	Res#8	Res#9	Res#10
Set #1	0	0	0	0						
Set #2		1	1	1	1					
Set #3			1	1	1	1				
Set #4				1	1	1	1			
Set #5					1	1	1	1		
Set #6						0	0	0	0	
Set #7							0	0	0	0
TOT	0	1	1	1	1	1	1	1	0	0

D

Case 3

	Res#1	Res#2	Res#3	Res#4	Res#5	Res#6	Res#7	Res#8	Res#9	Res#10
Set #1	1	1	1	1						
Set #2		0	0	0	0					
Set #3			0	0	0	0				
Set #4				0	0	0	0			
Set #5					0	0	0	0		
Set #6						0	0	0	0	
Set #7							1	1	1	1
TOT	1	1	1	1	0	0	1	1	1	1

Figure S6: Calculating the imperfect-helicity on an amylose chain of size 10. A) shows a schematic where each circle represents a glucose residue and each square bracket divided residues into different sets. The blue square brackets show sets #1 and #7, green sets #2 and #6, red sets #3 and #5, and black set #4. B-D) display three different cases from actual simulations for calculating the imperfect-helicity of the chain based on the imperfect-helicity of each set.

to frame.

This strictness is by design as we aim for a conservative estimation of helicity and thus prefer to reject some helix-like conformations than to allow random structures into the analysis. However, it also introduces a rapid helix-breaking mechanism that is purely an artifact of our criteria. These fluctuations are on the order of frame to frame and thus have minimal effect on characterizing imperfect helices.

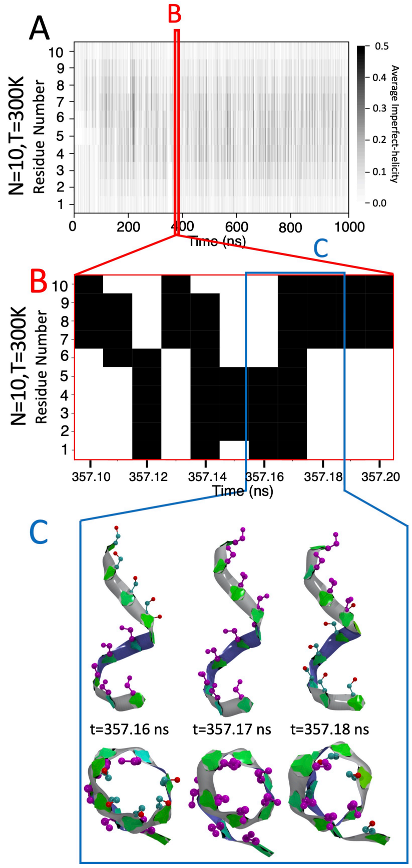

Figure S7: Short time fluctuations in helicity. A) shows a heat map of the average imperfect-helical structures for $N = 10$ at $T = 300$ K. Each data point is averaged over 100 frames, each 10 ps apart. B) displays a zoomed-in portion showing the period from 357.10 ns to 357.20 ns in detail. Black represents the residues in imperfect-helical structure, while white represents non-helical residues. Side and top view snapshots of selected three consecutive frames in B are shown in C). At $t = 357.16$ ns 50% of the chain is imperfect-helical, at $t = 357.17$ ns 100% of the chain is imperfect-helical and at $t = 357.18$ ns 40% of the chain is imperfect-helical. The C5–C6–O6 atoms for all glucose units in the chain are shown explicitly. Magenta indicates glucose units that are considered to be imperfect-helical.

5 Total Helicity of the Chain

The total helicity of the chain is calculated by simply counting the number of residues that are part of an imperfect-helix. Thus, this measurement does not differentiate between 2

imperfect-helices of length 4 and 1 imperfect-helix of length 8. The results for all 3 chain lengths are shown in figure S8.

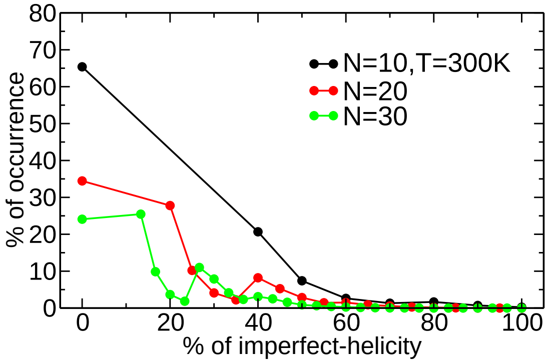

Figure S8: The probability of finding portions of the amylose chain in imperfect-helical structures for all three lengths at $T = 300$ K. The x-axis represents the portion of the chain in imperfect-helical structures, i.e., the sum of all individual structures.

As before, the probability of having no imperfect-helices decays significantly with increasing chain length. Similarly, the probability of finding longer helical sections decays rapidly with the length of the helix. However, for $N = 20$ and $N = 30$, there are clear peaks at certain percent helicity values. These peaks correspond to the probability of finding multiple shorter helices on the same chain. The largest peak occurs for finding 2 imperfect-helices of length 4 which occurs at 40% helicity for $N = 20$ and ∼26% for $N = 30$. There is also a small peak around 40% helicity for $N = 30$ which would correspond to finding 3 imperfect-helices of length 4.

References

(1) Jorgensen, W. L.; Chandrasekhar, J.; Madura, J. D.; Impey, R. W.; Klein, M. L. Comparison of simple potential functions for simulating liquid water. *The Journal of Chemical Physics* **1983**, *79*, 926–935, DOI: 10.1063/1.445869.

(2) Jorgensen, W. L.; Chandrasekhar, J.; Madura, J. D.; Impey, R. W.; Klein, M. L. Comparison of simple potential functions for simulating liquid water. *The Journal of chemical physics* **1983**, *79*, 926–935.
